# Supplementary material for: SARS-CoV-2 Recombination and Coinfection Events Identified in Clinical Samples in Russia
Source: Viruses. 2023 Jul 30;15(8):1660. doi: 10.3390/v15081660 (PMC10458065; doi:10.3390/v15081660)
Supplement: Supplementary file 1 [file viruses-15-01660-s001.zip › Supplementary methods.pdf]

Algorithm for the detection of recombinant genomes and coinfections

To search for potential recombinant viral genomes and coinfections cases, an algorithm was developed that allows separating sequencing data into 1) samples containing reads of a single variant of genome, 2) samples containing the genomes of several lines of the virus and being coinfections, 3) samples that are potential recombinant variants. The algorithm requires the database of nucleotide variants described above for its operation. To determine the phylogenetic lineage of the virus based on known nucleotide variants, the records in the database were filtered according to the following set of criteria:

- for every reference genetic lineage, nucleotide variations with the frequencies not exceed 0.9 were removed;
- nucleotide variations located at low coverage loci (10600-10650, 14406-14410, 21800-21850, 23020-23084, 29829-29879) and oligonucleotide annealing sites for viral genome amplification were removed.

Nucleotide variants from vcf files were filtered out using following parameters: DP<1000 and MQ<50, variants that were not in the Database of SARS-CoV-2 Nucleotide Variants were removed. SNVs detected in each sample were compared with the SNVs or InDels identified in each genetic lineage and their possible pairs. If the entire set of SNVs in a sample corresponds with any genetic lineage and all of substitutions are unambiguous (the sample contains only an alternative allele), then the sample is considered belong to one of genetic lineages in the database. Otherwise, the sample belong to either to unknown lineage or combination of lineages. The value of the similarity is calculated, depending on the number of matching and different SNVs. The calculations were made as follows: if the substitution is one of the defining one of the genetic lineages and is present in the considered sample, then this replacement is assigned the symbol "+"; if the substitution is present in the sample, but not one of the substitutions defining one of genetic lineages, then the symbol "-" is assigned; if the SNV is absent in the sample and is not related with genetic lineage, then the symbol "/" is assigned; if the substitution is associated with genetic lineage, but was not identified in the sample, then the symbol "*" is assigned. Thus, each nucleotide variation is assigned as pair of symbols (for example, "+-" or "*/") when comparing nucleotide sequences of two genetic lines. According to described algorithm, the most relevant pairs of genetic lineages, were suggested to genomes that were not identified as a single genetic lineage. Additionally, for each substitution, the presence of the reference and alternative alleles in the sample allows to identify cases of coinfection. So, if the symbolic designation of a substitution for a pair of strains is "+-", and both the reference and alternative alleles are present in the sample (0/1 in the .vcf file), then the similarity indicator of the considered substitution in the sample with the substitution for the pair of strains is assigned value 3, if only the alternative allele is present in the sample (1/1 in the .vcf file), then the value of the similarity index will be -1. It should be noted that the assigned values of the measure of similarity were selected empirically, a complete table of possible values depending on the symbolic representation of substitutions is presented in Table S2. The obtained values of similarity measures of substitutions are summarized for each pair of genetic lines, after which the pair with the highest value of the total similarity measure is selected.

If in all detected substitutions in the sample contain only the alternative allele (1/1 in the .vcf file) and none of them is assigned the symbolic designation "+-" or "-+", then the sample is considered to contain the genome of only one genetic line (suggested as single genotype). If the symbols contain the values "+-" or "-+", then it is considered that the genome of the sample is potentially recombinant (suggested as a recombination). If the sample contains substitutions with alternative and reference alleles, then it is considered that the sample contains the genomes of viruses of different genetic lines (suggested as coinfection).

Visualization and evaluation of results was performed using in-house algorithm to display allele frequencies for a selected pair of genetic lineages based on sequence reads alignments (.bam files). For suggested pairs of viral genetic lineages, substitutions are filtered in the database of nucleotide variants, as described above. Using the Python library pysam, the sample .bam file was accessed. For each position from the filtered list of substitutions, the frequency of each of the nucleotide was calculated. A final table was formed including coordinates of identified nucleotide substitutions and the frequency of reference and alternative alleles, which is visualized in a histogram obtained using Python library matplotlib.
