# Supplementary material for: SARS-CoV-2 Recombination and Coinfection Events Identified in Clinical Samples in Russia
Source: Viruses. 2023 Jul 30;15(8):1660. doi: 10.3390/v15081660 (PMC10458065; doi:10.3390/v15081660)
Supplement: Supplementary file 1 [file viruses-15-01660-s001.zip › Table S4.pdf]

Table S4. List of samples from singleton branches adjacent to LQ-24283 with indication of lineage.

Sample	GISAID	Line Nextclade
hCoV-19/Panama/SEQ1200-GMI/2022	XAF	XAF
hCoV-19/Venezuela/Car9661/2022	BA.2	XAF
hCoV-19/Algeria/22459/2022	XY	XY
hCoV-19/Panama/SEQ1185- GMI/2022	BA.2	XAB
hCoV-19/Panama/M246747-GMI/2022	BA.2	XAB
hCoV-19/Guatemala/5131-LNS/2022	BA.2	XAB
hCoV-19/Panama/SEQ1147-GMI/2022	BA. 2	XAB
hCoV-19/Colombia/DC-IAVH-VG-18756/2022	BA.2	XAB
hCoV-19/DominicanRepublic/1996371-LNSPDD/	BA.2	XAG
